# Deduction of Novel Genes Potentially Involved in Osteoblasts of Rheumatoid Arthritis Using Next-Generation Sequencing and Bioinformatic Approaches

**DOI:** 10.3390/ijms18112396

**Published:** 2017-11-11

**Authors:** Yi-Jen Chen, Wei-An Chang, Ya-Ling Hsu, Chia-Hsin Chen, Po-Lin Kuo

**Affiliations:** 1 Graduate Institute of Clinical Medicine, College of Medicine, Kaohsiung Medical University, Kaohsiung 807, Taiwan; chernkmu@gmail.com (Y.-J.C.); 960215kmuh@gmail.com (W.-A.C.); 2 Department of Physical Medicine and Rehabilitation, Kaohsiung Medical University Hospital, Kaohsiung 807, Taiwan; 3 Department of Physical Medicine and Rehabilitation, Kaohsiung Municipal Ta-Tung Hospital, Kaohsiung 801, Taiwan; 4 Division of Pulmonary and Critical Care Medicine, Kaohsiung Medical University Hospital, Kaohsiung 807, Taiwan; 5 Graduate Institute of Medicine, College of Medicine, Kaohsiung Medical University, Kaohsiung 807, Taiwan; hsuyl326@gmail.com; 6 Department of Physical Medicine and Rehabilitation, School of Medicine, College of Medicine, Kaohsiung Medical University, Kaohsiung 807, Taiwan; 7 Orthopaedic Research Center, Kaohsiung Medical University, Kaohsiung 807, Taiwan; 8 Institute of Medical Science and Technology, National Sun Yat-Sen University, Kaohsiung 804, Taiwan

**Keywords:** rheumatoid arthritis, bone erosion, osteoblasts, next-generation sequencing, bioinformatics, microRNA, messenger RNA

## Abstract

The role of osteoblasts in peri-articular bone loss and bone erosion in rheumatoid arthritis (RA) has gained much attention, and microRNAs are hypothesized to play critical roles in the regulation of osteoblast function in RA. The aim of this study is to explore novel microRNAs differentially expressed in RA osteoblasts and to identify genes potentially involved in the dysregulated bone homeostasis in RA. RNAs were extracted from cultured normal and RA osteoblasts for sequencing. Using the next generation sequencing and bioinformatics approaches, we identified 35 differentially expressed microRNAs and 13 differentially expressed genes with potential microRNA–mRNA interactions in RA osteoblasts. The 13 candidate genes were involved mainly in cell–matrix adhesion, as classified by the Gene Ontology. Two genes of interest identified from RA osteoblasts, A-kinase anchoring protein 12 (*AKAP12*) and leucin rich repeat containing 15 (*LRRC15*), were found to express more consistently in the related RA synovial tissue arrays in the Gene Expression Omnibus database, with the predicted interactions with miR-183-5p and miR-146a-5p, respectively. The Ingenuity Pathway Analysis identified *AKAP12* as one of the genes involved in protein kinase A signaling and the function of chemotaxis, interconnecting with molecules related to neovascularization. The findings indicate new candidate genes as the potential indicators in evaluating therapies targeting chemotaxis and neovascularization to control joint destruction in RA.

## 1. Introduction

Rheumatoid arthritis (RA) is an autoimmune disease characterized by systemic inflammation, presence of autoantibodies, and targeted synovitis, affecting approximately 0.5–1% of population [1]. Articular manifestation of inflammatory arthritis is the hallmark of RA and a major determinant of the disease activity [2]. Numerous cell types are involved in the pathophysiology of RA, including immune cells like T cell, B cells and macrophages, synoviocytes, and chondrocytes [3,4]. Synoviocytes and chondrocytes are cell types within the joint dominantly affected by RA. Activated synovial fibroblasts within the inflamed synovium have altered morphology and behavior, and attach directly to cartilage and release matrix degradation enzymes, leading to the destruction of cartilage tissue [1,5,6]. They also interact indirectly with adjacent macrophages through the release of receptor activator of nuclear factor κB ligand (RANKL) and mediate the differentiation of macrophage precursors into osteoclasts [6,7].

Bone erosion is a characteristic feature of affected joints in RA, known to be triggered mainly by synovitis, producing pro-inflammatory cytokines and RANKL [8]. Within the inflamed joint structure, the destructive process by the pannus, a structure formed by proliferative synovium containing infiltrates of immune cells, proliferative vessels, and increased osteoclasts, leads to bone erosion particularly at the synovium–bone interface where the pannus invades [9]. Through the direct contact of the invading pannus to the bone and increased angiogenesis within the pannus structure, the capacity of the synovial fibroblasts to damage structures within the joint has been widely proposed [8,9,10,11]. Together with synovial fibroblasts and immune cells, the critical role of osteoclasts in the disrupted bone homeostasis under pathological condition such as RA is being studied. The imbalance of bone homeostasis in RA leads to peri-articular bone loss, which usually precedes bone erosion and further progression of joint destruction [12].

While osteoclasts are the major cells responsible for bone loss and bone erosion in RA, the impaired differentiation and function of osteoblasts have been proposed in recent studies, and inflammatory tissue in RA may impair osteoblast activity, which respond differently from osteoblasts of osteoarthritic condition [12,13,14,15]. The precursors of immune cells are formed and maintained in the bone marrow, where osteoclasts and osteoblasts reside and act to maintain a balanced bone remodeling; therefore, research on the interplay between the immune system and the skeletal system has emerged to gain more knowledge on the novel field termed osteoimmunology [8,16,17].

MicroRNAs (miRNAs) are non-coding single strand RNAs consisting of 20–22 nucleotides, acting primarily on the 3’UTR of mRNAs to regulate gene expressions through a post-transcriptional manner [18]. These small non-coding RNAs participate in the regulation of numerous cellular processes and dysregulation of miRNAs are associated with various diseases [19]. The role of miRNA regulation in bone diseases has been reported, including osteoporosis and arthritis that are associated with altered bone homeostasis [20,21]. The study of gene associations using the next generation sequencing (NGS) technique and bioinformatics approaches has evolved, providing high-throughput genomic profiling and further understanding and analysis of functional annotations of identified genes and/or miRNAs [22,23]. In this study, we used various bioinformatics tools and databases to assist in identifying potential miRNA–mRNA interactions in osteoblasts of RA population, including miRmap [24], Gene Expression Omnibus (GEO) [25], Ingenuity^®^ Pathway Analysis (IPA) [26], and Database for Annotation, Visualization and Integrated Discovery (DAVID) [27].

The role of osteoblasts in the development of peri-articular bone loss and limited repair of bone erosion in RA has received much attention, and miRNAs are hypothesized to play critical roles in the regulation of osteoblast function in RA. The aim of our current study is to explore novel miRNAs differentially expressed in osteoblasts of RA bone and to identify genes potentially involved in the dysregulated bone homeostasis in RA. Using the NGS for genomic profiling and various bioinformatics approaches, we expect the findings will provide novel insights into potential therapeutic targets that contribute to better control of RA disease activity.

## 2. Results

### 2.1. Identification of Differentially Expressed miRNAs and Potential miRNA–mRNA Interactions between Osteoblasts of Normal and Rheumatoid Arthritis Bones

**Figure 1:**
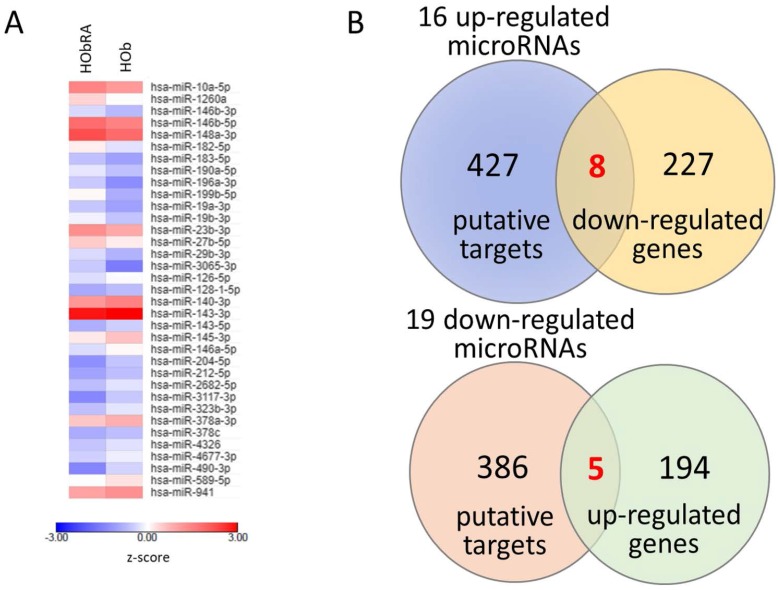
Identification of differentially expressed microRNAs and potential microRNA–mRNA interactions in rheumatoid arthritis primary osteoblasts. (**A**) The next generation sequencing (NGS) identified 35 differentially expressed microRNAs (thresholds of >2.0-fold change and reads per million (RPM) >10) in rheumatoid arthritis (RA) osteoblasts, compared to normal osteoblasts. The heat map analysis with z-score values is shown here. (**B**) The 16 up-regulated and 19 down-regulated microRNAs predicted 435 and 391 putative targets, respectively, using the miRmap database with selection threshold of miRmap score ≥99.0. Additionally, 434 protein-coding genes with >2.0-fold change and >0.3 fragments per kilobase of transcript per million (FPKM) were identified from the NGS, where 199 genes were up-regulated and 235 genes were down-regulated in RA osteoblasts. The putative targets of up-regulated (down-regulated) microRNAs were matched to the down-regulated (up-regulated) protein-coding genes by the Venn diagram analysis. Finally, thirteen genes (eight down-regulated and five up-regulated) with potential miRNA–mRNA interactions were identified.

To identify differentially expressed miRNAs and mRNAs between osteoblasts of normal and RA bones, and potential miRNA–mRNA interactions involved in the inflammatory process and bone homeostasis, we simultaneously performed RNA-seq and small RNA-seq by NGS of normal osteoblasts and RA osteoblasts. There were 35 miRNAs with >2.0-fold change and >10 reads per million (RPM) of either origin of osteoblasts identified. Sixteen up-regulated and 19 down-regulated miRNAs in RA osteoblasts compared to normal osteoblasts were identified, as listed in Table 1. Figure 1A presents the heat map analysis of the 35 differentially expressed miRNAs with z-score values. In addition, we found 434 protein-coding genes with >2.0-fold change and >0.3 fragments per kilobase of transcript per million (FPKM), where 199 genes were up-regulated and 235 genes were down-regulated in RA osteoblasts, compared to normal osteoblasts. To determine potential miRNA–mRNA interactions in normal and RA osteoblasts, we first analyzed putative targets of the 35 miRNAs by miRmap database, and selected targets with miRmap score of more than 99.0. The results yielded 435 targets (520 interactions) of 16 up-regulated miRNAs and 391 targets (477 interactions) of 19 down-regulated miRNAs. We then matched these predicted targets by up-regulated (down-regulated) miRNAs to down-regulated (up-regulated) genes from the 434 protein-coding genes selected. By Venn diagram analysis, we identified 13 genes (eight down-regulated genes and five up-regulated genes) with potential miRNA–mRNA interactions in RA osteoblast (Figure 1B).

### 2.2. The 13 Candidate Genes Were Involved in the Cell Matrix Adhesion and Related Molecular Functions

**Figure 2:**
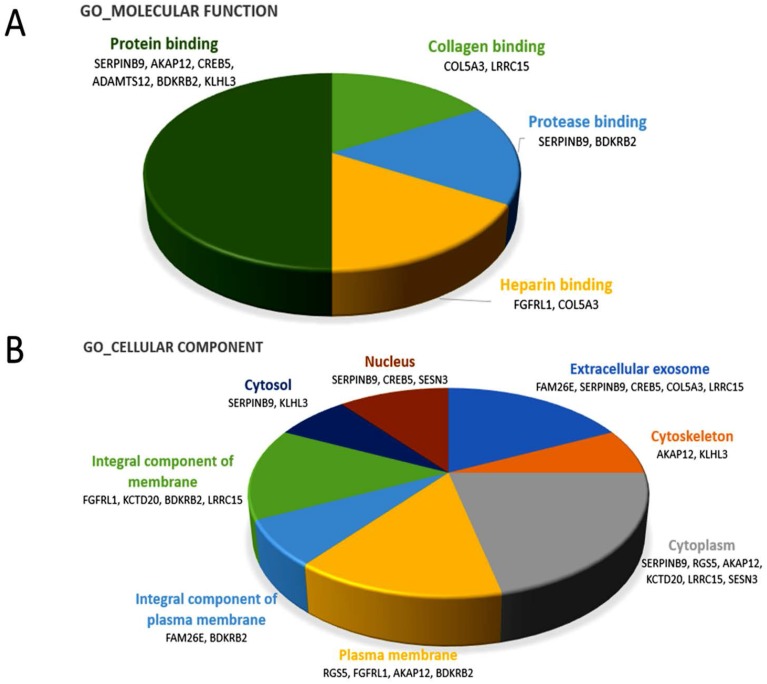
Gene ontology terms involved in the 13 candidate genes of rheumatoid arthritis primary osteoblasts. Using the functional annotation analysis in the DAVID database, the 13 candidate genes were classified by terms of the Gene Ontology in: the molecular function domain (**A**); and the cellular component domain (**B**). The related genes are listed below each term. The selected criteria for functional annotation analysis was EASE = 1.0.

The 13 candidate genes identified from normal and RA osteoblasts were analyzed by DAVID database to determine the functional annotations of these genes. Setting the EASE threshold at 1.0, the Gene Ontology classified these genes to be involved in cell–matrix adhesion and G-protein coupled receptor signaling pathway in the domain of biological process. In the domain of molecular function, the genes were involved in the bindings of collagen, protease, heparin and protein, as shown in Figure 2A. In the domain of cellular component, the genes were mostly active in the extracellular exosome, cytoskeleton, cytoplasm, and plasma membrane (Figure 2B). The KEGG pathway identified *CREB5*, *BDKRB2* and *COL5A3* to be involved in the cGMP-PKG and PI3K-Akt signaling pathways.

### 2.3. Analysis of Candidate Genes from Normal and Rheumatoid Arthritis (RA) Osteoblasts in Gene Expression Omnibus (GEO) Database and Identification of Potential Molecular Signatures in RA Joint Microenvironment

**Figure 3:**
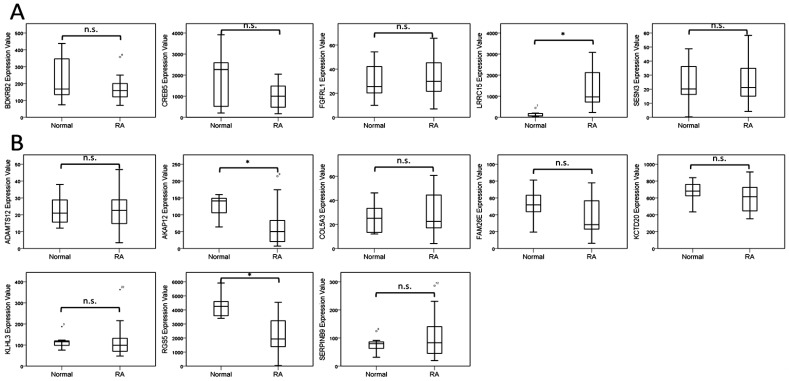
Analysis of 13 genes with potential microRNA–mRNA interactions in the Gene Expression Omnibus (GEO) database. The expression values of: five up-regulated genes (**A**); and eight down-regulated genes (**B**) identified from normal and rheumatoid arthritis (RA) osteoblasts were validated in a representative array (GSE77298) of normal and RA synovial tissues from the GEO database. Significant up-regulation of *LRRC15* and down-regulation of *AKAP12* and *RGS5* were observed in the synovial tissues of patients with RA, compared to the normal subjects. * indicated *p* < 0.05, and n.s. indicated no statistical significance. (Probe ID reference: *BDKRB2*, 205870_at; *CREB5*, 229228_at; *FGFRL1*, 223321_s_at; *LRRC15*, 213909_at; *SESN3*, 242899_at; *ADAMTS12*, 221421_s_at; *AKAP12*, 231067_s_at; *COL5A3*, 218975_at; *FAM26E*, 230254_at; *KCTD20*, 228299_at; *KLHL3*, 221221_s_at; *RGS5*, 209071_s_at; and *SERPINB9*, 242814_at).

The 13 candidate genes with potential miRNA–mRNA interactions identified from normal and RA osteoblasts are listed in Table 2. To determine the involvement of these genes in the joint microenvironment of RA patients, we searched in the GEO database for RA related arrays. There were no arrays comparing osteoblasts or bones of normal and RA patients available in the GEO database. However, we found five arrays comparing synovial tissues of normal and RA patients (GSE1919, GSE55235, GSE55475, GSE7307 and GSE77298), one array comparing synovial fibroblasts isolated from synovial tissues of normal and RA patients (GSE29746) and two arrays comparing synovial macrophages of normal and RA patients (GSE10500 and GSE97779). We analyzed the expression values of the 13 candidate genes identified from normal and RA osteoblasts in these RA related arrays and found two genes that were expressed in the same direction in RA synovial tissues more consistently. As shown in Table 3, the up-regulated *LRRC15* in RA osteoblasts was found up-regulated in four out of the five arrays of synovial tissues; the down-regulated *AKAP12* in RA osteoblasts was found down-regulated in four out of the five arrays of synovial tissues; and up-regulated in one array of synovial macrophages. Additionally, the up-regulated *CREB5* and down-regulated *KCTD20* in RA osteoblasts were observed to express in the same direction in arrays of synovial macrophages. The expression values of the 13 candidate genes in the representative array dataset (GSE77298) are shown in Figure 3.

### 2.4. Potential miRNA–mRNA Interactions of LRRC15 and AKAP12 in RA Osteoblasts

**Figure 4:**
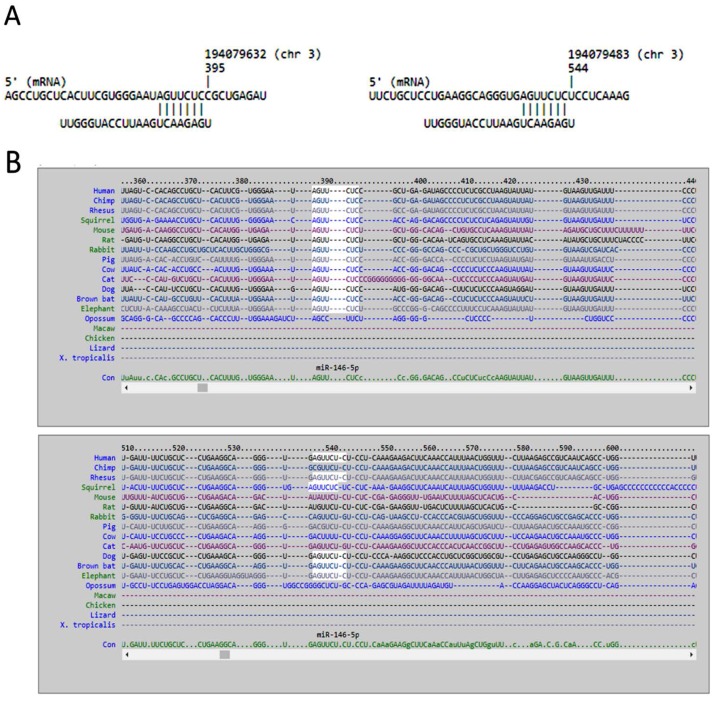
The putative binding sites of miR-146a-5p on *LRRC15*. The sequences and putative binding sites of miR-146a-5p on the 3′UTR of *LRRC15* at positions of 388–394 and 537–543 were validated in: miRmap (**A**); and TargetScan (**B**).

**Figure 5:**
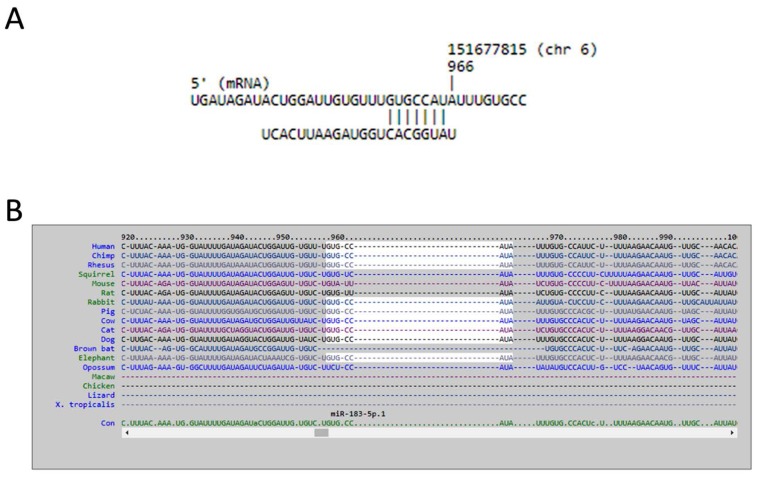

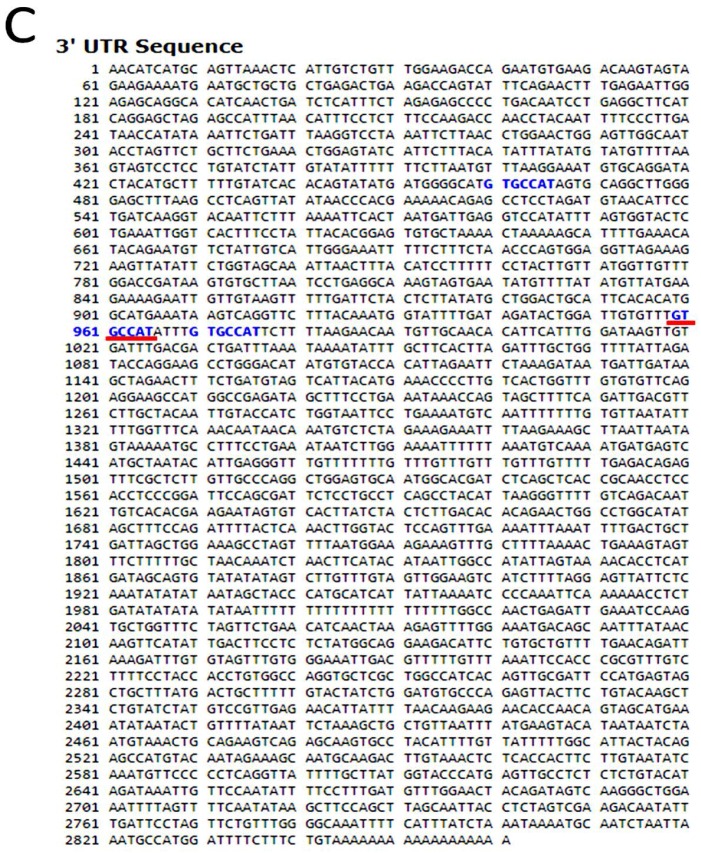
The putative binding site of miR-183-5p on *AKAP12*. The sequence and putative binding site of miR-183-5p on the 3′UTR of *AKAP12* at the position of 959–966 was validated in: miRmap (**A**); TargetScan (**B**); and miRDB (**C**).

We used miRmap database to analyze potential miRNA regulations of *LRRC15* and *AKAP12*. Forty-four miRNAs with miRmap score >99.0 were potentially involved in the *LRRC15* regulation, and 11 miRNAs with miRmap score >99.0 were potentially involved in the *AKAP12* regulation. Matching to our differentially expressed miRNA database, we identified down-regulated miR-146a-5p that potentially up-regulated *LRRC15*, and up-regulated miR-183-5p that potentially down-regulated *AKAP12*, as shown in Table 4. The sequences and putative 3′UTR binding sites of representative miRNAs in *LRRC15* and *AKAP12* were then validated in miRmap, TargetScan and miRDB databases. The target binding sites of miR-146a-5p in the 3′UTR of *LRRC15* at the positions of 388–394 and 537–543 were validated in miRmap and TargetScan (Figure 4), while the target binding site of miR-183-5p in the 3′UTR of *AKAP12* at the position of 959–966 was validated in miRmap, TargetScan and miRDB (Figure 5).

**Figure 6:**
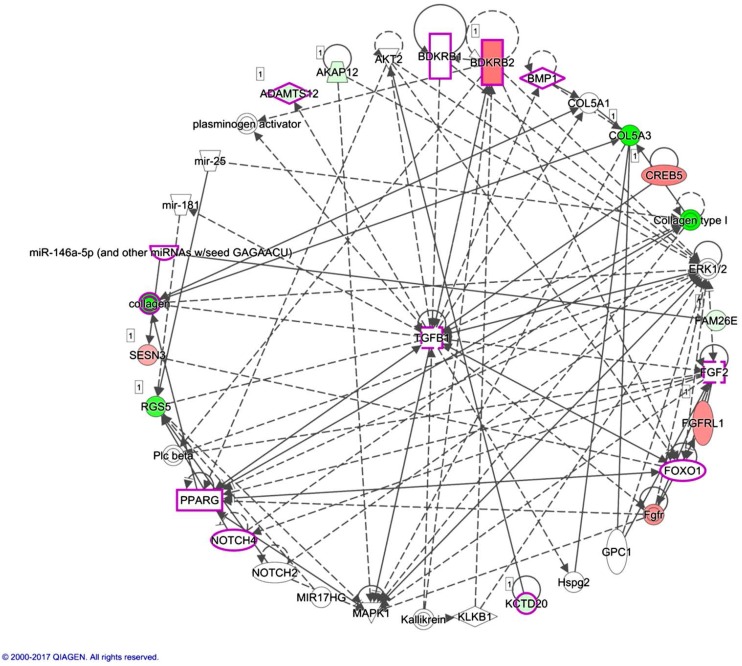
Network analysis by Ingenuity^®^ Pathway Analysis (IPA) indicated molecules associated with rheumatic disease and inflammation of joint. The network analysis was performed by IPA software to indicate networks involved in the 13 candidate genes from rheumatoid arthritis (RA) osteoblasts. Ten of the 13 candidate genes were grouped in one network associated with cardiovascular system development and function, cellular development, cellular growth and proliferation. Molecules including *ADAMTS12*, *BDKRB1*, *BDKRB2*, *BMP1*, *FGF2*, *FOXO1*, *KCTD20*, *NOTCH4*, *PPARG*, *TGFB1* and miR-146a-5p were associated with rheumatic disease and inflammation of joint in the network, as indicated in purple frames. Molecules in green indicated down-regulated expressions, and molecules in red indicated up-regulated expressions in RA osteoblasts compared to normal osteoblasts. The green and red color scales disclosed the relative gene expression values of RA to normal osteoblasts.

Using the IPA software, diseases and functions associated with the 13 candidate genes identified between normal and RA osteoblasts were further analyzed. There were two networks classified, as shown in Table 5, with 10 of the 13 candidate genes involved in network 1 (Figure 6). The previously identified *AKAP12* was involved in network 1, while *LRRC15* was involved in network 2. One of the miRNAs identified between normal and RA osteoblasts, miR-146a-5p, was also involved in network 1. Using the overlay diseases and functions tool in the IPA software, we disclosed *ADAMTS12*, *BDKRB1*, *BDKRB2*, *BMP1*, *FGF2*, *FOXO1*, *KCTD20*, *NOTCH4*, *PPARG*, *TGFB1* and miR-146a-5p to be associated with “rheumatic disease” and “inflammation of joint” in network 1, as indicated by purple frames in Figure 6.

In addition to network analysis, we found miR-29b-3p to be one of the significant upstream regulators (*p*-value of overlap = 3.94 × 10^−4^), which was 2.51-fold up-regulated in RA osteoblasts compared to normal osteoblasts. The effectors potentially involved in the miR-29b-3p regulation were *BDKRB2*, *COL5A3*, *CREB5*, *KCTD20*, and *SERPINB9* (Table 6).

### 2.5. The Differentially Expressed Genes in RA Osteoblasts Were Associated with Chemotaxis, Neovascularization, Cell Adhesion and Extracellular Matrix Organization

To identify pathways and biological functions involved in RA osteoblasts, the 434 differentially expressed protein-coding genes (199 up-regulated genes and 235 down-regulated genes) between normal and RA osteoblasts were further analyzed by the IPA software and the functional annotation tool in the DAVID database. The results of IPA analysis identified *TGFB1*, *TNF*, *IFNG*, and *IL1B* among the top upstream regulators. miR-29b-3p was also a significant upstream regulator (*p*-value of overlap = 1.76 × 10^−5^), with its effectors listed in Table 6. The differentially expressed genes were categorized into 25 networks. *AKAP12*, along with 24 other dysregulated genes were involved in diseases and functions related to cellular development, cellular growth and proliferation, and organ development, whereas 17 dysregulated genes, including *LRRC15*, were involved in diseases and functions related to hereditary disorder, immunological disease, and organismal injury and abnormalities (Table 7).

**Figure 7:**
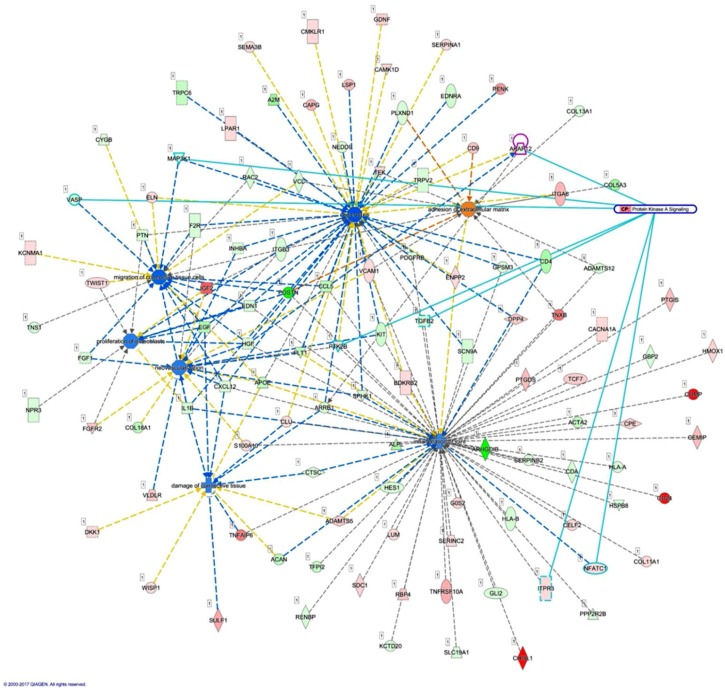
Analysis of the interconnection between *AKAP12* and the merged networks of related joint diseases and functions. The 434 differentially expressed genes identified in normal and rheumatoid arthritis (RA) osteoblasts were analyzed by the IPA to be categorized into 25 networks. Diseases and functions related to joint destruction in RA microenvironment, including inflammation of joint, chemotaxis, damage of connective tissue, migration of connective tissue cells, proliferation of osteoblasts, and neovascularization were selected to identify related genes. *AKAP12*, one of the genes involved in chemotaxis, was connected to *HGF* and *ARRB1*, molecules involved in damage of connective tissue and neovascularization. In addition, the overlay canonical pathway analysis indicated *AKAP12*, *MAP3K1*, *VASP*, *PTK2B*, *TGFB2*, *ITPR3*, and *NFATC1* (marked in light blue) to be involved in the protein kinase A signaling, and participated in various indicated networks.

We then determined the interconnection between various diseases and functions, including inflammation of joint, chemotaxis, damage of connective tissue, migration of connective tissue cells, proliferation of osteoblasts, and neovascularization. *AKAP12* is one of the genes involved in chemotaxis, having connection with *HGF* and *ARRB1*, which are involved in damage of connective tissue and neovascularization. Using the overlay canonical pathway tool in the IPA, molecules involved in protein kinase A signaling, including *AKAP12*, *MAP3K1*, *VASP*, *PTK2B*, *TGFB2*, *ITPR3*, and *NFATC1*, also take part in the above selected diseases and functions, as indicated by light blue lines in Figure 7.

**Figure 8:**
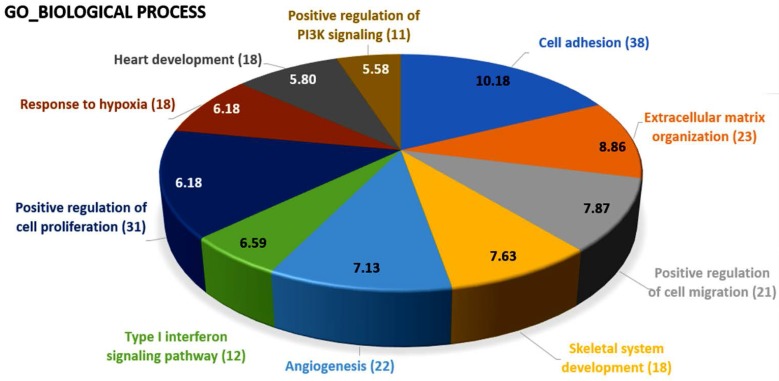
The biological process analysis of differentially expressed genes in rheumatoid arthritis osteoblasts. The 434 differentially expressed genes in rheumatoid arthritis osteoblasts were analyzed in the DAVID database for the identification of involved biological processes. The results indicated these genes were potentially involved in cell adhesion (38 genes), extracellular matrix organization (23 genes), positive regulation of cell migration (21 genes), skeletal system development (18 genes), angiogenesis (22 genes), type I interferon signaling pathway (12 genes), positive regulation of cell proliferation (31 genes), response to hypoxia (18 genes), heart development (18 genes), and positive regulation of PI3K signaling (11 genes). The selected criteria for functional annotation analysis were EASE = 0.1 and fold enrichment >1.3. The proportions of the pie chart were drawn according to the numbers of genes involved in each biological term, and the numbers within the pie chart indicated −log (*p*-value) of each biological term.

Using the DAVID database for the analysis of biological functions, the top 10 biological functions involved in these differentially expressed genes of RA osteoblasts were cell adhesion (38 genes), extracellular matrix organization (23 genes), positive regulation of cell migration (21 genes), skeletal system development (18 genes), angiogenesis (22 genes), type I interferon signaling pathway (12 genes), positive regulation of cell proliferation (31 genes), response to hypoxia (18 genes), heart development (18 genes), and positive regulation of PI3K signaling (11 genes), as shown in Figure 8.

## 3. Discussion

The current study identified 35 differentially expressed miRNAs and 13 candidate genes potentially involved in osteoblasts of RA, using NGS and bioinformatics analysis. Two of the 13 candidate genes differentially expressed in osteoblasts of RA, *LRRC15* and *AKAP12*, were found to have consistent direction of expression in the synovial tissue of RA patients identified in four of the five RA array datasets (GSE7307, GSE55475, GSE77298, GSE55235, and GSE1919). The potential miRNA regulation on *LRRC15* was miR-146a-5p, whereas the potential miRNA regulation on *AKAP12* was miR-183-5p, predicted by miRmap, TargetScan and miRDB database. We proposed the novel findings of miR-146a-5p–*LRRC15* and miR-183-5p–*AKAP12* regulations in the altered function of osteoblasts in RA microenvironment.

The role of osteoblasts in the development of bone loss and limited capacity of repair of bone erosion in RA has received more attention recently, and miRNAs are hypothesized to play critical roles in the regulation of the function of osteoblasts in RA [13,15,28]. The results of IPA analysis identified miR-29b-3p to be a potential upstream regulator of the differentially expressed genes in RA. The regulation of the miR-29 family is proposed to participate in osteoarthritis and cartilage homeostasis [29]. In addition, the miR-29 family has been shown to promote osteoblast differentiation by targeting inhibitors of the Wnt signaling pathway, and to possess numerous distinct activities at different stages of osteoblast differentiation. In the mature osteoblasts, miR-29 targets collagen type I, reducing the rate of collagen synthesis and facilitating structural stability of the bone [30]. In a review article by Miao et al., the Wnt signaling pathway participates in the pathogenesis of RA and bone remodeling. They suggested that inhibition of the Wnt signaling pathway may contribute to impaired osteoblast function in RA, and increased expression of several inhibitors of the Wnt signaling pathway may contribute to bone resorption in RA [31]. Two of the effectors of miR-29b-3p in our candidate genes, *BDKRB2* and *SERPINB9*, were categorized into the molecular function of protease binding by gene ontology, as shown in Figure 2A and Table 6, which may support the involvement of miR-29b-3p in the regulation of bone matrix in RA.

A-kinase anchoring protein 12 (AKAP12), one of the A-kinase anchoring proteins, is a scaffold protein for protein kinase A (PKA) and protein kinase C (PKC) that control cytoskeleton dynamics, cell migration, and cell adhesion [32,33]. Studies suggested that the down-regulation of AKAP12 induces the formation of stress fibers and proliferation of adhesion complexes; increased cellular senescence was also observed in AKAP12-null mice [34]. The findings potentially link *AKAP12* to the disrupted bone homeostasis in RA, where the study by Yudoh and colleagues revealed the higher rate of cellular senescence and greater decline in the replicative capacity of peri-articular osteoblasts in RA patients, compared to osteoarthritic patients [15]. The expressions of AKAP12 in inflammatory responses were studied. The increased protein expression of AKAP12 in the fibrotic scar may restrict excessive inflammation during central nervous system repair [35], and decreased protein levels of AKAP12 were observed in the lung tissue of patients with chronic obstructive pulmonary disease [36], suggesting the participation of AKAP12 in the regulation of inflammatory response. There is not much literature discussing the role of AKAP12 in the bone homeostasis or the inflamed joint microenvironment. One study identified *Akap12* to be one of the genes possibly involved in the alternative splicing in bone following mechanical loading in rat model [37]. The altered joint structures along with inflamed peri-articular soft tissue in RA predispose the affected joint to increased mechanical loading, which may disrupt the PKA-mediated mechanotransduction.

Scaffold proteins serve as connecting hubs that modulate both upstream signaling molecules and the downstream effectors within cells. The expression and activity of AKAP12 is proposed to be affected by the hypoxic tumor microenvironment [38]. Studies also suggested *AKAP12* to be a tumor suppressor and angiogenesis suppressor gene that down-regulates vascular endothelial growth factor, potentially through epigenetic regulation [39]. The role of miRNA regulation in altered bone homeostasis has been reported [20,21]. Few studies also reported that miR-183 increased osteoclastogenesis through the binding on the 3′UTR of heme oxygenase-1 [40], and oxidative stress within the bone marrow microenvironment may alter miRNA cargo of extracellular vesicles, expressing high abundance of miR-183 cluster and miR-183-5p transfection inhibits the osteogenic differentiation of bone marrow stromal cells [41]. Altogether, with these literature reviews, the up-regulated miR-183-5p with its putative target of down-regulated *AKAP12* in our NGS result suggests the novel finding of potential miR-183-5p–*AKAP12* regulation in the changed bone homeostasis in RA joint microenvironment.

Leucine rich repeat containing 15 (*LRRC15*), also named *LIB*, is a gene encoding leucine-rich transmembrane protein that participates in the cell–matrix adhesion and cell migration, and is induced and highly expressed in various cancer types [42,43,44]. The role of *LRRC15* in inflammation is also proposed, as it is induced by beta-amyloid and pro-inflammatory cytokines in astrocytes of Alzheimer's disease brain [45,46] and up-regulated by pro-inflammatory stimuli during the process of dental caries [47]. There is still lack of related literature on the role of *LRRC15* in the arthritic joint microenvironment or other bone diseases.

miR-146a-5p was identified to be one of the molecules associated with the inflammation of joint in the IPA analysis, as indicated in Figure 6. miR-146 is one of the miRNAs strongly implicated in RA, regulating a group of target genes related to inflammation. The level of miR-146a is increased in synovial tissue, synovial fluid, whole blood and many other cells types such as T cells, B cells and macrophages. However, the association between the expression level of miR-146a and disease activity is still inconclusive [48]. miR-146a is also proposed to prevent joint destruction in arthritic mice by inhibiting osteoclastogenesis [49]. Our results indicated the potential regulation of miR-146a-5p on *LRRC15*. Whether the regulation of miR-146a-5p in bone is mediated by inflammatory joint microenvironment or other stimuli merits further clarification.

**Figure 9:**
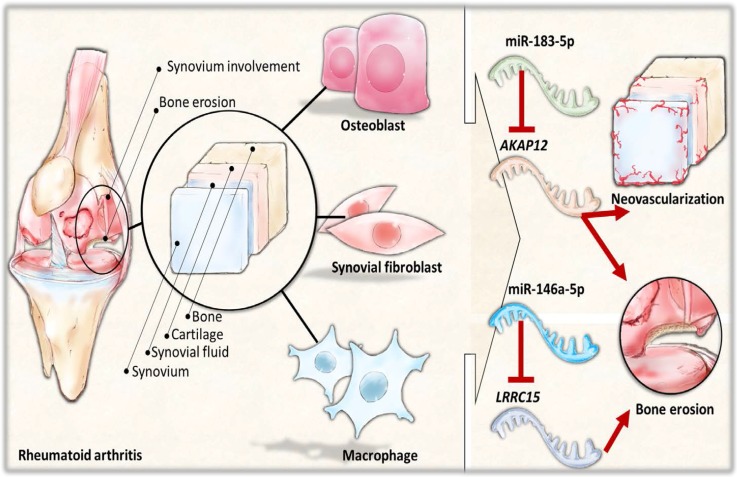
The proposed novel molecular signatures and microRNA regulations in rheumatoid arthritis osteoblasts.

The interconnection between different cell types and the arthritic microenvironment is complex yet important in the understanding of the RA disease entity. The area of pannus formation is infiltrated by synoviocytes, immune cells, osteoclasts and proliferative vessels, and comes into direct contact with adjacent bone surface, where osteoblasts reside [9]. Along with immune cells and synovial fibroblasts, the role of osteoblasts in the pathogenesis of articular destruction in RA have gained much attention, with the function of osteoblasts being compromised at sites of focal erosion and reduced mineralization of the newly formed bone in the arthritic joints [50]. The activity and function of osteoblasts is also inhibited by the hypoxia within the arthritic joint microenvironment [51]. In the current study, we explored novel miRNAs and the putative targets expressed in the osteoblasts of RA origin. Several of the target genes identified in our study were also found to have consistent directions of expression in the synovial tissues of RA patients from related GEO array datasets. The schematic potential molecular mechanisms are summarized in Figure 9. These identified miRNA–mRNA regulations may provide novel perspectives into deeper understanding of the cell–cell communication between different cell types within the joint structure and the role of arthritic joint microenvironment in the altered bone homeostasis.

## 4. Materials and Methods

### 4.1. Primary Cell Culture

Osteoblasts isolated from normal human bones (HOb, Catalog No. 406-05a, Lot No. 3145) and bones of patients with RA (HObRA, Catalog No. 406RA-05a, Lot No. 1796) were purchased from Cell Applications, Inc. (San Diego, CA, USA). In detail, osteoblasts of normal bone were obtained from a 66-year-old female, and osteoblasts of RA bone were obtained from a 72-year-old female with RA. Osteoblasts were grown in human osteoblast growth medium (Cell Applications, Inc.) and maintained in 37 °C incubator containing 5% CO_2_ until confluence. The HOb and HObRA cells were then harvested for total RNA extraction and further mRNA and small RNA profiling.

### 4.2. RNA Sequencing

To prepare samples for mRNA and small RNA profiling, total RNAs from HOb and HObRA cells were extracted by Trizol^®^ Reagent (Invitrogen, Carlsbad, CA, USA) according to the manufacturer’s instructions. Before further sequencing, the quality of extracted RNAs were analyzed by OD_260_ detection using ND-1000 spectrophotometer (Nanodrop Technology, Wilmington, DE, USA) and validated by RNA integrity number (RIN) with Agilent Bioanalyzer (Agilent Technology, Santa Clara, CA, USA), where RINs for HOb and HObRA were 9.9 and 10, respectively. Total RNA sequencing analysis for RNA-seq and small RNA-seq were performed by Welgene Biotechnology Company (Welgene, Taipei, Taiwan). We set the criteria for differentially expressed mRNAs and miRNAs at fold change >2.0, FPKM >0.3 for mRNA and RPM >10 for miRNA.

### 4.3. miRmap Database

miRmap is an open-source database that provides miRNA target prediction using a comprehensive approach with various computational tools, including thermodynamic, evolutionary, probabilistic and sequence-based approaches. The repression strength of a miRNA–mRNA interaction of interest is indicated by the miRmap score. The higher miRmap score indicates higher repression strength. The 35 differentially expressed miRNAs were consecutively inputted to obtain putative target genes, and those with miRmap scores higher than 99.0 were selected for further analysis [24].

### 4.4. Gene Expression Omnibus (GEO)

The GEO database provides public access to high-throughput array- and sequence-based data. The dataset of interest also provides link to web-based tool such as GEO2R and users can look for candidate genes and perform further analysis by obtaining raw data with expression values of specific genes in the array [25]. The arrays related to joint tissues of RA patients (GSE1919, GSE55235, GSE55475, GSE7307, GSE77298, GSE29746, GSE10500 and GSE7779) were used in this study to identify genes that expressed in consistent directions with our NGS results.

### 4.5. Ingenuity^®^ Pathway Analysis (IPA)

The Ingenuity^®^ Pathway Analysis (IPA) software (Ingenuity systems, Redwood City, CA, USA) contains large database with detailed and structured findings reviewed by experts which was derived from thousands of biological, chemical and medical researches, and provide researchers with quick searching. The IPA also enables analysis, integration, and recognition of data from gene and SNP arrays, RNA and small RNA sequencing, proteomics and many other biological experiments; in addition, deeper understanding and identification of related signaling pathways, upstream regulators, molecular interactions, disease process and candidate biomarkers are also available [26].

### 4.6. DAVID Database

The DAVID database is a bioinformatics resource that assists in the analysis of a list of genes derived from high-throughput genomic sequencing experiments, using different tools such as functional annotation and gene functional classification. The analysis results help researchers gain overall understanding of the involved terms of gene ontology, signaling pathways and diseases within the genes of interest [27].

### 4.7. Statistical Analysis

The expression values of target genes obtained from arrays of GEO database were analyzed using IBM SPSS Statistics for Windows, version 19 (IBM Corp., Armonk, NY, USA). To compare the differences of expression values between normal and RA groups, non-parametric method with Mann-Whitney U test was used. A statistically significant difference was determined by *p*-value < 0.05.

## 5. Conclusions

Our study indicates that miR-183-5p–*AKAP12* and miR-146a-5p–*LRRC15* regulations participate in the altered function of osteoblasts in RA joint microenvironment, which are partly responsible for the pathogenesis of bone erosions. The current findings suggest new candidate genes as potential indicators in evaluating therapies targeting chemotaxis and neovascularization to control joint destruction in RA.

## Figures and Tables

**Table 1:** Differentially expressed miRNAs in normal and rheumatoid arthritis (RA) osteoblasts.

microRNA	Precursor	HObRA Seq (Norm)	HOb Seq (Norm)	Fold Change
hsa-miR-3065-3p	hsa-mir-3065	14.08	1.26	11.17
hsa-miR-199b-5p	hsa-mir-199b	55.42	7.36	7.53
hsa-miR-196a-3p	hsa-mir-196a-2	14.34	2.31	6.21
hsa-miR-148a-3p	hsa-mir-148a	12,452.28	4404.25	2.83
hsa-miR-19a-3p	hsa-mir-19a	13.43	4.83	2.78
hsa-miR-19b-3p	hsa-mir-19b-1	35.6	14.08	2.53
hsa-miR-29b-3p	hsa-mir-29b-1	22.17	8.83	2.51
hsa-miR-23b-3p	hsa-mir-23b	1117.61	471.84	2.37
hsa-miR-182-5p	hsa-mir-182	67.94	28.69	2.37
hsa-miR-146b-5p	hsa-mir-146b	4319.58	1843	2.34
hsa-miR-183-5p	hsa-mir-183	11.08	4.83	2.29
hsa-miR-10a-5p	hsa-mir-10a	1708.04	752.73	2.27
hsa-miR-1260a	hsa-mir-1260a	126.48	56.64	2.23
hsa-miR-190a-5p	hsa-mir-190a	28.56	13.24	2.16
hsa-miR-146b-3p	hsa-mir-146b	21.38	10.61	2.02
hsa-miR-27b-5p	hsa-mir-27b	160.78	80.29	2.00
hsa-miR-490-3p	hsa-mir-490	1.43	21.12	−14.77
hsa-miR-3117-3p	hsa-mir-3117	1.56	15.87	−10.17
hsa-miR-204-5p	hsa-mir-204	2.22	14.82	−6.68
hsa-miR-143-3p	hsa-mir-143	54,069.61	16,0924.3	−2.98
hsa-miR-143-5p	hsa-mir-143	6.39	18.92	−2.96
hsa-miR-212-5p	hsa-mir-212	4.3	12.51	−2.91
hsa-miR-323b-3p	hsa-mir-323b	10.95	31.53	−2.88
hsa-miR-2682-5p	hsa-mir-2682	10.3	29.63	−2.88
hsa-miR-146a-5p	hsa-mir-146a	22.69	64.84	−2.86
hsa-miR-145-3p	hsa-mir-145	77.45	212.17	−2.74
hsa-miR-140-3p	hsa-mir-140	814.97	1900.06	−2.33
hsa-miR-4326	hsa-mir-4326	12.65	28.69	−2.27
hsa-miR-4677-3p	hsa-mir-4677	16.69	37.1	−2.22
hsa-miR-128-1-5p	hsa-mir-128-1	5.35	11.66	−2.18
hsa-miR-378a-3p	hsa-mir-378a	174.47	376.1	−2.16
hsa-miR-126-5p	hsa-mir-126	23.47	50.02	−2.13
hsa-miR-378c	hsa-mir-378c	5.87	12.4	−2.11
hsa-miR-941	hsa-mir-941-1	516.36	1049.39	−2.03
hsa-miR-589-5p	hsa-mir-589	49.16	98.36	−2.00

**Table 2:** Candidate genes identified from putative targets of microRNAs and differentially expressed genes between normal and RA osteoblasts.

Gene Symbol	Gene Name	Fold-Change (HObRA/HOb)
*BDKRB2*	bradykinin receptor B2	3.20
*CREB5*	cAMP responsive element binding protein 5	3.11
*FGFRL1*	fibroblast growth factor receptor-like 1	2.76
*LRRC15*	leucine rich repeat containing 15	5.85
*SESN3*	sestrin 3	2.17
*ADAMTS12*	ADAM metallopeptidase with thrombospondin type 1 motif 12	0.37
*AKAP12*	A-kinase anchoring protein 12	0.39
*COL5A3*	collagen, type V, alpha 3	0.07
*FAM26E*	family with sequence similarity 26 member E	0.46
*KCTD20*	potassium channel tetramerization domain containing 20	0.32
*KLHL3*	kelch like family member 3	0.38
*RGS5*	regulator of G-protein signaling 5	0.11
*SERPINB9*	serpin family B member 9	0.32

**Table 3:** Analysis of 13 candidate genes in RA related arrays in Gene Expression Omnibus (GEO) datasets.

Accession #	GSE7307	GSE55475	GSE77298	GSE55235	GSE1919	GSE29746	GSE10500	GSE97779
Specimen	Synovial tissue					Fibroblast	Synovial macrophage	
Numbers	N/RA	N/RA	N/RA	N/RA	N/RA	N/RA	N/RA	N/RA
	5/5	10/13	7/16	10/10	5/5	11/9	3/5	5/9
**Up-regulated mRNA**								
*BDKRB2*	n.s.	n.s.	n.s.	DOWN	n.s.	n.s.	UP	n.s.
***CREB5***	n.s.	n.s.	n.s.	n.s.	n.s.	n.s.	**UP**	**UP**
*FGFRL1*	n.s.	--	n.s.	--	--	n.s.	--	n.s.
***LRRC15***	n.s.	**UP**	**UP**	**UP**	**UP**	n.s.	n.s.	n.s.
*SESN3*	UP	--	n.s.	--	--	n.s.	--	n.s.
**Down-regulated mRNA**								
*ADAMTS12*	n.s.	n.s.	n.s.	n.s.	--	n.s.	--	DOWN
***AKAP12***	**DOWN**	n.s.	**DOWN**	**DOWN**	**DOWN**	n.s.	UP	n.s.
*COL5A3*	UP	n.s.	n.s.	n.s.	--	n.s.	--	n.s.
*FAM26E*	n.s.	--	n.s.	--	--	n.s.	--	n.s.
***KCTD20***	n.s.	**DOWN**	n.s.	n.s.	n.s.	n.s.	**DOWN**	**DOWN**
*KLHL3*	DOWN	n.s.	n.s.	UP	--	n.s.	--	n.s.
*RGS5*	n.s.	n.s.	DOWN	n.s.	n.s.	n.s.	UP	DOWN
*SERPINB9*	n.s.	n.s.	n.s.	n.s.	UP	n.s.	n.s.	UP

The genes and their directions of expression marked in **bold** were those that were expressed more consistently in the same directions in RA synovial tissues from GEO datasets. N, normal population; RA, rheumatoid arthritis patients; UP, up-regulated in RA; DOWN, down-regulated in RA; n.s., non-significant between normal and RA; --, no identical probes within the array.

**Table 4:** Potential miRNA regulations of corresponding predicted targets.

**Down-Regulated miRNA**	**Precursor**	**Fold-Change**	**miRmap Score**	**Predicted Target Up-Regulated mRNA**	**Fold-Change (HObRA/HOb)**
hsa-miR-146a-5p	hsa-mir-146a	−2.86	99.08	*LRRC15*	5.85
**Up-Regulated miRNA**	**Precursor**	**Fold-Change**	**miRmap Score**	**Predicted Target Down-Regulated mRNA**	**Fold-Change (HObRA/HOb)**
hsa-miR-183-5p	hsa-mir-183	2.29	99.56	*AKAP12*	0.39

**Table 5:** Networks associated with 13 candidate genes differentially expressed in RA osteoblasts.

	Top Diseases and Functions	Score	Focus Molecules	Molecules in Network
1.	Cardiovascular System Development and Function, Cellular Development, Cellular Growth and Proliferation	27	10	↓**ADAMTS12**, ↓**AKAP12**, AKT2, BDKRB1, ↑**BDKRB2**, BMP1, COL5A1, ↓**COL5A3**, collagen, Collagen type I, ↑**CREB5**, ERK1/2, ↓**FAM26E**, FGF2, Fgfr, ↑**FGFRL1**, FOXO1, GPC1, Hspg2, Kallikrein, ↓**KCTD20**, KLKB1, MAPK1, mir-25, mir-181, miR-146a-5p, MIR17HG, NOTCH2, NOTCH4, plasminogen activator, Plc beta, PPARG, ↓**RGS5**, ↑**SESN3**, TGFB1
2.	Cellular Compromise, Organismal Injury and Abnormalities, Hereditary Disorder	6	3	AKT2, COASY, DOLPP1, FOSB, FOSL2, FURIN, GBP2, H2AFB3, IGSF8, JUND, KEAP1, ↓**KLHL3**, ↑**LRRC15**, MAFG, MAPK3, MARK4, miR-3656, miR-423-5p, miR-4537, miR-6825-5p, MRPS27, NOS3, P2RY8, PLEKHM1, PRKCG, RAB11B, SELPLG, ↓**SERPINB9**, SLC9A1, SMARCD1, TAGLN, UNC119, USF2, WNK2, WNK4

The genes marked in **bold** were the 13 candidate genes identified in normal and RA osteoblasts.

**Table 6:** Upstream regulator miR-29b-3p and potential downstream effectors of RA osteoblasts.

**Analysis**	**Molecules in 13 Candidate Genes**	***p*-Value of Overlap**
miR-29b-3p	*BDKRB2*, *COL5A3*, *CREB5*, *KCTD20*, *SERPINB9*	3.94 × 10^−4^
**Analysis**	**Molecules in 434 Differentially Expressed Genes**	***p*-Value of Overlap**
miR-29b-3p	*RUNX1T1*, *PALM*, *PDPN*, *LAMA2*, *ITGA6*, *CEMIP*, *DPP4*, *HS3ST3B1*, *KIF26B*, *CACNA1A*, *GPR85*, *CELF2*, *HAPLN1*, *BDKRB2*, *NDN*, *CRYBG1*, *CREB5*, *WISP1*, *FAM167A*, *SLC12A8*, *COL11A1*, *DMKN*, *MEGF6*, *ENPP2*, *ID3*, *PDGFRB*, *RTL5*, *TRAF5*, *LASP1*, *CSPG4*, *HAPLN3*, *NEDD9*, *KCTD20*, *SERPINB9*, *PEG10*, *UACA*, *ADAM19*, *CTPS1*, *CCDC85A*, *FCRLA*, *TRPC6*, *COL5A3*, *CCDC81*, *HEYL*	1.76 × 10^−5^

**Table 7:** Two of the networks associated with 434 candidate genes differentially expressed in RA osteoblasts.

Top Diseases and Functions	Score	Focus Molecules	Molecules in Network
Cellular Development, Cellular Growth and Proliferation, Organ Development	38	25	↓**AKAP12**, Cbp/p300, ↓**CDA**, ↑**CEMIP**, ↓**CNN1**, ↑**CRLF1**, ↓**DOCK10**, E2f, EGLN, ↑**EPHA4**, ↓**EPHA5**, ↑**FGFRL1**, ↓**FHL1**, ↓**FLT1**, GTPase, ↓**GUCY1B3**, Hedgehog, ↓**HGF**, ↑**ICA1**, Importin alpha, ↑**ITGA6**, ↑**ITGB8**, ↓**LMOD1**, ↓**MYOCD**, ↓**NOTCH1**, ↓**PDLIM3**, ↑**PHLDA1**, ↑**PLXNA2**, Proinsulin, ↓**SCUBE3**, Sfk, Smad2/3, ↑**SOX9**, Vegf, ↑**VLDLR**
Hereditary Disorder, Immunological Disease, Organismal Injury and Abnormalities	22	17	↑**ADM2**, ATP6AP1, ATP6V1F, CASP2, CDK2AP2, ↑**CMKLR1**, CTSA, ↓**CYGB**, ↑**ELFN1**, ↓**FAM46B**, FOXRED2, GPR84, GRM4, ↓**HAAO**, ↓**HEYL**, ↓**IRX2**, ↓**KCTD20**, ↑**KIF26B**, LDB1, ↑**LRRC15**, ↓**LRRC32**, ↓**LYPD1**, miR-4656, miR-504-3p, ↑**MKX**, ↓**MX1**, PLPPR2, ↓**PLPPR4**, PPP1CA, RASSF8, ↓**SERPINA9**, TBC1D22A, TRIM67, TUFT1, ZNF677

The genes marked in **bold** were the 434 differentially expressed protein-coding genes identified in normal and RA osteoblasts.

## Abbreviations

AKAP12	A-Kinase Anchoring Protein 12
DAVID	Database for Annotation, Visualization and Integrated Discovery
FPKM	Fragments per Kilobase of Transcript per Million
GEO	Gene Expression Omnibus
HOb	Osteoblasts Isolated from Normal Human Bones
HObRA	Osteoblasts Isolated from Rheumatoid Arthritis Bones
IPA	Ingenuity^®^ Pathway Analysis
LRRC15	Leucine Rich Repeat Containing 15
miRNAs	microRNAs
NGS	Next Generation Sequencing
PKA	Protein Kinase A
RA	Rheumatoid Arthritis
RANKL	Receptor Activator of Nuclear Factor κB Ligand
RPM	Reads per Million
